# Market Structure, Financial Dependence and Industrial Growth: Evidence from the Banking Industry in Emerging Asian Economies

**DOI:** 10.1371/journal.pone.0160452

**Published:** 2016-08-04

**Authors:** Habib Hussain Khan, Rubi Binit Ahmad, Chan Sok Gee

**Affiliations:** Department of Finance and Banking, Faculty of Business and Accountancy, University of Malaya, Kuala Lumpur, Malaysia; Universidad Veracruzana, MEXICO

## Abstract

In this study, we examine the role of market structure for growth in financially dependent industries from 10 emerging Asian economies over the period of 1995–2011. Our approach departs from existing studies in that we apply four alternative measures of market structure based on structural and non-structural approaches and compare their outcomes. Results indicate that higher bank concentration may slow down the growth of financially dependent industries. Bank competition on the other hand, allows financially dependent industries to grow faster. These findings are consistent across a number of sensitivity checks such as alternative measures of financial dependence, institutional factors (including property rights, quality of accounting standards and bank ownership), and endogeneity consideration. In sum, our study suggests that financially dependent industries grow more in more competitive/less concentrated banking systems. Therefore, regulatory authorities need to be careful while pursuing a consolidation policy for banking sector in emerging Asian economies.

## Introduction

The significance of financial institutions for economic growth is well established in economics and finance literature. Several channels through which financial institutions may contribute to economic well-being have been documented. For example, financial institutions play a key role in providing information and allocating resources by evaluating firms’ prospects and devoting resources to promising ventures [1]; they take on a risk-sharing role by financing mega-projects with high returns accompanied by high risk [2]; and they perform a monitoring function over borrowers [3]. Once the role of financial institutions for economic activities has been recognized, the level of bank competition/concentration becomes relevant for many reasons. First, it can influence banks’ efficiency, the product quality and the extent of invention/innovation [4–9]. Second, the linkage between bank concentration/competition and economic stability is also relevant to financial institutions [10–13]. Third, bank concentration/competition can also affect firms’ access to credit and the monetary policy transmission [14–17]. However, literature with respect to role of competition/concentration for economic growth is still in its early stages and only a handful of studies have so far explored this relationship. Even these studies are limited in scope, for three important reasons. First, they are far from reaching a consensus. Second, their analysis covers a pre-financial crisis period which may not be applicable to post-crisis times because of changing competitive conditions triggered by mergers and acquisitions that occurred in response to the Asian Financial Crisis 1997–1998 and Global Financial Crisis 2008–2009. Third, their analysis is based on a single measure of competition, which could be misleading. These limitations are discussed one by one below.

With respect to the first limitation, the literature provides two contradictory findings. First, more concentrated/less competitive banking systems negatively affect the economic growth of financially dependent industries, while such industries grow more in less concentrated/more competitive banking systems. (See for example, [4, 18, 19]). Second, the concentrated banking markets actually promote economic growth while higher level of competition suppresses economic growth. (See for example [20–26]).

With respect to the second limitation, the competitive conditions in Asian banking markets have changed substantially in recent years. These changes can be attributed to bank consolidations, privatization, financial integration, deregulation, and financial reforms in response to the global financial crisis of 2008–2009 [27–29]. These developments raise serious concerns regarding the desirability of bank concentration or competition for industrial growth owing to an ambiguous competition-growth relationship.

Regarding the third limitation, there is an important debate with respect to the measure of banking market competition. Under the structure-conduct-performance paradigm (SCP), concentration is negatively related to the level of competition. [30] demonstrate that a high level of concentration in banking is likely to reduce the competition. However [31] and [32] indicate that even highly concentrated markets can be competitive due to information asymmetries. Similarly, [33] show that a higher level of concentration and competition both enhance the banking system stability and reduce the probability of crisis, and their findings thus provide indirect evidence of a positive relationship between competition and concentration.

Moreover, a frequently used measure of competition, the Panzar-Rosse (PR) model [34], has been criticized for a number of reasons, including its inability to measure the level of competition/market power. (See [35], pp. 26–27, for a detailed discussion on the disadvantages of the PR model). The Lerner Index [36] is yet another measure of market power/level of competition; however, it also suffers from weaknesses. In contrast, the Boon Indicator [37] has emerged as a better measure of competition, as it avoids the major econometric and theoretical drawbacks of the PR model and Lerner Index. Though some authors may favor any competition measure, there is general disagreement among researchers with respect to the best measure. According to [38], inferences about the level of competition differ widely using different indicators of banking market competition, and hence the implications of competition depend upon the choice of indicators. Using only one measure of market structure can thus be misleading, because each measure captures a unique aspect of competition and has its own advantages and disadvantages. Therefore, it is more effective to use several measures of market structure.

In order to address the above issues, we apply structural and non-structural measures of market structure and relate them to the growth of externally financially dependent industries in 10 Asian emerging markets (China, Hong Kong, India, Indonesia, Japan, Korea, Malaysia, Philippines, Singapore and Thailand). We also consider the role of financial development (bank and capital market development) and other factors that might explain the banking structure-growth relationship. These factors include growth opportunities as well as institutional factors such as property rights, quality of accounting standards and bank ownership.

This study contributes towards finance literature in general and bank literature in particular in several aspects. First, it compares the findings from both structural and non-structural measures in order to have a better understanding of the competition-growth relationship. Second, it uses recent data that covers both the Asian Financial Crisis 1997–1998 and the Global Financial Crisis 2008–2009. Third, it considers the banking industry in emerging Asian economies where the literature on the topic is almost non-existent. Thus, four measures of banking market structures are used in this study: two structural ones–the five-bank concentration ratio (CR5) and the Herfindahl Hirschman Index (HHI)–and two non-structural ones–the Boon Indicator (BI) and the Lerner Index (LI)–and the results are compared. The data from 10 emerging Asian economies over the period of 1995–2011 are analyzed. The results show that less concentrated and more competitive banking systems boost the growth of externally financially dependent industries.

The rest of the study is organized as follows. A review of the relevant literature is presented in Section 2, while Section 3 addresses model development and measurement of variables. In Section 4 the estimation results are reported on and discussed, and finally, the conclusion and policy implications are reported in Section 5.

## Literature Review

Earlier evidence on the role of financial institutions for economic growth comes from [1], who argue that good financial systems boost economic growth by enhancing the probability of successful innovations. On the other hand, any disruption in the financial sector hampers the innovation process, leading to a reduction in overall economic growth. Similarly, [39] show that even after accounting for political and other economic factors, the economic growth is higher for economies with a higher level of bank development and stock market development. An influential study by [40] is the foundation of research in the domain of bank development, financial dependence of industries and economic growth. The authors estimate the external financial dependence of manufacturing firms by using firm level data and show that countries with a more developed financial market experience greater industrial growth ([40]. A few other studies which highlight the role of financial sector development for economic growth include [41], [42] and [43].

Thus the importance of financial institutions for economic growth is well recognized in the literature. However, the role of bank market structure (competition/concentration) for economic growth is still in the early stages. The few studies which look into this domain are far from reaching a consensus and provide two seemingly contradictory views: one favoring a higher level of competition/lower level of concentration for economic growth while the other suggests the opposite, as shown below.

According to the first view, firms’ growth is limited in highly concentrated or less competitive banking systems because firms have less access to finance. The limited growth of firms (due to a lack of easy access to credit) translates into overall lower economic growth [18, 19]. [44] on the other hand find that a higher level of bank concentration negatively affects the industrial and per capita income growth, but that this relationship is significant only for low-income countries. The underlying logic for this view is that competitive banking systems make access to finance easy and affordable for firms, enabling them to borrow and invest more. [45] and [46] support this view and demonstrate that concentrated/less competitive banking systems result in low firm creation and as a result less economic growth. [47] argue that banks operating in concentrated markets tend to use their market power and charge high loan rates which make funding more expensive for firms, and expensive funding depresses firms’ investing activities. Similarly, [4] provide evidence that industries that are more dependent on external finance grow more in a more competitive banking environment.

The second view is that banks in more concentrated markets perform the function of information producer and establish a strong relationship with their customers. On the other hand, increased competition can lead to asymmetry of information between borrowers and lenders, less lending and less investment [24]. Moreover, [22] argues that banking competition hampers the screening role performed by banks in their choice of borrowers. Banks in competitive markets take less care in screening firms and also charge higher loan rates. A higher cost of borrowing decreases the availability of funds. According to [21] and [25], economies with less competitive markets experience more creation and emergence of new firms. Evidence supporting this view also comes from [20], who show that the growth of industries that are dependent on external finance is faster in economies with concentrated banking systems.

A few recent contributions highlighting the favorable effects of concentration for economic growth come from [48], [49] and [50]. [48] argues that financially dependent industries in concentrated banking markets perform better than those operating in more competitive markets. [49] show that a higher level of concentration in the banking market increases the overall growth of the manufacturing industry. However, the effect is stronger for industries with a small firm size, a lower incorporation rate, and less dependence on public debt (and, hence, relatively greater reliance on banks). In contrast, [50] find that both competition and concentration measures are positively related to economic growth, which may indicate that measures of concentration do not necessarily represent a low level of competition.

To summarize, the overall evidence on the role of banking structure (competition/concentration) for economic growth is ambiguous and provides little policy inputs as to whether concentration or competition is favorable for economic growth. Furthermore, only a few studies on competition-growth–[4] and [48]–use the Panzar-Rosse model (a non-structural approach) along with structural approaches (CR5 and HHI). However, research has shown that the PR model is subject to several theoretical and econometric drawbacks, including its inability to measure level of competition/market power. The Boon Indicator, on the other hand, is a better measure and avoids the major econometric and theoretical drawbacks of the PR model. This study addresses some of the issues of earlier studies by using several measures for both concentration and competition: CR5, HHI, the Lerner Index and the Boone Indicator.

## Methodology

To determine the role of the banking market structure for industrial growth in emerging Asian markets, the methodology is the one applied by earlier studies, such as [20], [4], [48]. First the measure of industrial growth on market structure measures (concentration/competition) is regressed and then the measures of bank development and stock market development (collectively referred to as financial development), and financial dependence as in [20], are introduced.

### Basic Model

In this study a basic model has been developed that examines the effect of concentration/competition on industrial growth in general; that is, without considering industry characteristics e.g. financial dependence.

$${\mathrm{Growth}\left(Value\;Added\right)}_{jct}=\alpha +\beta_{1}{Market\;Structure}_{ct}+\beta_{2}{Market\;Capitalization}_{ct}+\beta_{3}{Domestic\;Credit\;to\;Private\;Sector}_{ct}+\gamma_{1}{Country\;Controls}_{c}+\gamma_{2}{Industry\;Dummies}_{j}+{\gamma_{3}Time}_{t}+\epsilon_{jct}$$ (1)

Where subscripts *j*, *c* and *t* respectively indicate industries, countries and time. Growth (Value Added) is the annual growth of industry value added. Industry, country and time fixed effects are included so as to capture unobservable heterogeneity across industries, countries and time respectively. Following [20], included here are the measure of stock market development (market capitalization), measure of bank development (domestic credit to private sector), and country level controls such as per capital GDP, and an Index of quality of accounting standards to address the misspecification issues. The Market Structure variable represents the measures of bank concentration and bank competition, to be discussed in detail in the next section. As mentioned in the introduction, the expected signs on coefficient of market structure is ambiguous.

### Extended Model

The basic model identifies the overall impact of competition/concentration on industrial growth. In order to decompose this effect at country and industry levels, an extended model is constructed in which two interaction terms are included. First, between the market structure measures (concentration and competition) and industries’ financial dependence; and second, between the financial dependence of industry and financial development.

$${\mathrm{Growth}\left(Value\;Added\right)}_{jct}=\alpha +\beta_{1}[{Market\;Structure}_{ct}]+\beta_{2}[{External\;Dependence}_{]j}]+\beta_{3}[{Financial\;Development}_{ct}]+\beta_{4}\left[Market\;Structure_{ct}*External\;Dependence_{]j}\right]+\beta_{5}{\left[External\;Dependence_{j}*Financial\;Development\right]}_{ct}+\gamma_{1}Industry\;Dummies_{j}+\gamma_{2}Country\;Controls_{c}+\gamma_{3}Time_{t}+\epsilon_{jct}$$ (2)

The interaction term of market structure and financial dependence tests whether financially dependent industries grow more/less in economies with high/low bank concentration/competition. The sign on *β*_4_ is not clear a priori owing to ambiguous evidence from the relevant literature. Interaction between external dependence and financial development is included, following earlier studies, to determine whether the growth of financially dependent industries is higher for economies with a well-developed financial sector. The sign on *β*_5_ is expected to be positive because this relationship has been extensively discussed in [40], and almost all subsequent studies in this domain endorse its positive relationship with industrial growth.

### Data and Variables

#### Industrial Growth

Data on variables used in the analysis by this study come from various sources, and the definitions of variables and sources of data are shown in Table 1. Data on the dependent variables in this study (annual growth in value added of manufacturing industries in each country) come from UNIDO (United Nations Database on Industrial Statistics).

**Table 1:** Definition and Source of variables.

Variable	Description
Industry Value Added	Industry Value Added represents the contribution of industry to overall gross domestic product (GDP). Data on value added for each manufacturing sector in each sample country has been obtained from the United Nations Database on Industrial Statistics.
Growth in Value Added	Annual growth rate in industry value added over the period of 1995–2011.
Share in Industry Value Added	Fraction of industry value added in overall value added of manufacturing sector over the period
Financial Dependence	External financial dependence has been extracted from Table 1 in [40] and Table 2 in [53]. (1) A dummy variable which equals 1 if financial dependence of a sector is above the median value and 0 otherwise. It thus bisects the data into two groups i.e. sectors located above mean value are highly dependent on external finance and those located below the median are less financially dependent. (2) Ranking of industries in groups of 4, and 10 in order of their financial dependence. The higher the rank the higher the financial dependence.
Private Credit	Credit provided to the private sector by the financial sector divided by GDP. Source: World Development Indicators of the World Bank.
Market Capitalization	Stock Market Capitalization divided by GDP. Source: World Development Indicators of the World Bank.
Total Capitalization	Sum of private credit and market capitalization.
Concentration	Total assets held by five largest banks of a country to the total assets of all banks in that country (5-bank concentration ratio). Sum of squared market shares of all the banks in each country (HHI). Source: Global Financial Database World Bank (for CR5), calculated based on banks’ total assets, obtained from BankScope.
Competition	The Lerner Index is the ratio of mark up (difference between out price and marginal cost) to output price. The higher values of Lerner indicate more market power and less competitive conditions. The Boone Indicator captures the reallocation of market share from inefficient to efficient firms. The stronger the effect (i.e. the larger the β in absolute value), the stronger the competition.
Accounting Standards	Accounting Standards is an index representing the quality of firms’ disclosure for a country. This index ranges from 0 to 90 with higher values indicating more disclosure. Source: Centre for International Financial Analysis and Research (CIFAR).
Property Rights Index	The Property Rights Index measures the enforcement of property rights. The index ranges from 0 to 100. Higher values of the index represent greater enforcement of property rights and hence greater protection. Source: Heritage Foundation.
GDP per capita	Log of per capita GDP over the period of 1995–2011. Source: World Development Indicators of the World Bank.

Note: Table shows the names, definitions and sources of the variables used in this study. Names are given in the first column, while in the second column provides a brief description of the variables and the sources from which data on these variables is collected.

#### Market Structure

For market structure, four different measures have been applied here: the 5-Bank concentration ratio (CR5), Hirschman Herfindahl Index (HHI), Lerner Index and Boon Indicator. Two of these measures (CR5 and HHI) are based on the structural approach from traditional Industrial Organization (IO) literature. Under this approach, the level of competition is inferred from the structure of the market (level of concentration). CR5 is measured as the fraction of total assets held by the five largest banks of a country over the total assets of all banks in that country. HHI is defined as the sum of squared market shares based on the assets of all the banks in each country. Both these measures have been used in literature to study the role of bank concentration for industrial growth (see [20]; [4]; [48]; and [49]). Data on CR5 has been obtained from the Global Financial Database of the World Bank while HHI has been calculated on the basis of the banks’ total assets, collected from Bankscope. The structural approach has been criticized for its inability to measure the true level of competition. Therefore, we also use two non-structural measures (i.e. the Lerner Index and Boone Indicator), from the New Empirical Industrial Organization (NEIO). The aim of the NEIO measures is to assess the level of competition directly from firms’ conduct.

#### Lerner Index

The Lerner Index directly measures the degree of market power. It is calculated as the ratio of mark-up to price of output:

$$Lerner_{i,t}=\left(Price_{i,t}-MC_{i,t}\right)/Price_{i,t}$$

Where *Price*_*i*,*t*_ is the price of the total assets and *MC*_*i*,*t*_ is the marginal cost of producing an additional unit of output. According to [36], the value of the Lerner Index ranges between 0 –indicating the state of no market power (perfect competition)–and 1 –indicating a situation of high market power (monopoly). Therefore, the higher values of Lerner indicate more market power and less competitive conditions.

#### Boone Indicator

The idea behind the Boone Indicator [37] is that efficient firms are highly rewarded and inefficient firms are more harshly punished in perfectly competitive markets. The Boone Indicator captures the market share transmission from inefficient to efficient firms. Thus the intensity of competition is measured from a profitability equation as follows:

$$ln\pi_{i}=\alpha +\beta lnc_{i}+\varepsilon_{i}$$

Where *π*_*i*_ and *c*_*i*_ represent bank profit and costs respectively. For banks with lower marginal costs, the profits are higher, therefore β < 0. Thus, increases in competition raise the profits of more efficient banks relative to less efficient ones. Larger values of β in absolute terms indicate higher levels of competition [51]. The data on the Lerner Index and Boone Indicator have been compiled from a variety of sources. The main source is the dataset of [52]. However, their data covers the period 1997–2010, and so we have collected data for the years 1995, 1996 and 2011 from Economic Research database of Federal Reserve Bank of St. Louis and Global Financial Development Database of World Bank.

#### External Financial Dependence

The financial dependence of an industry refers to the need for firms to raise finances from external sources; in other words, banks and/or capital markets. [40] determine the external dependence of the US manufacturing industry by using firm-level data. They define external financial dependence as the ratio of capital expenditure not financed with cash flows from operations to total capital expenditure. Almost all subsequent studies (for example, [20]; [4]; [53]; and [48]) use their data to infer the financial dependence of manufacturing sectors in other countries (See footnote 4 in [48] and footnote 6 in [4]). Due to mismatches in the sample period, we are unable to directly use their data. However, we use the ranking order of external financial dependence from Table 1 in [40] and Table 2 in [53]. We generate a dummy variable which equals 1 if financial dependence of a sector is above the median value and 0 otherwise. It thus bisects the data into two groups: that is, sectors located above the mean value are highly dependent on external finance and those located below the median are less financial dependent. For a robustness check we also rank industries in 4 and 10 groups in order of their financial dependence. However, the results from alternative rankings and dummy variables are qualitatively similar.

#### Financial Development

We follow [4] and use total capitalization as the measure of financial development. Total capitalization is the sum of stock market capitalization as a percentage of GDP which proxies for capital market development and domestic credit to the private sector as a percentage of GDP which represents bank development. Both stock market capitalization and private credit have also been used separately for a robustness check. Data on market capitalization and domestic credit to the private sector has been obtained from the World Development Indicators of the World Bank.

A few other variables, such as investment opportunities, property rights, accounting standards and bank ownership have been used for a robustness check. These variables and their sources are explained when they are used in estimation.

## Empirical Results

### Descriptive Statistics and Correlation Analysis

This section provides country wise descriptive account of variables of the study. Country wise averages, standard deviations, and minimum and maximum values on important variables are reported in Table 2. CR5 and HHI both represent the level of bank concentration in each country. Singapore is at the top in bank concentration, with an average value of CR at 0.962, followed by Korea with an average value of CR at 0.934. The Indian market is the least concentrated, with a CR of 0.433, while Japan and Hong Kong have the second and third least concentrated markets with an average CR of 0.497 and 0.498 respectively. For the rest of the countries, CR ranges between 0.661 for Thailand and 0.781 for Philippine, with only minor variations. The ranking of countries with respect to market concentration is not same with HHI, however. Singapore has the largest average for HHI (0.37), followed by China with an average value of HHI at 0.178. India, Japan and Hong Kong occupy the last three positions, with HHI at 0.087, 0.099 and 0.104 respectively.

**Table 2:** Descriptive Statistics.

Description	VA Growth	Industry Share	Log(VA)	Market Cap/GDP	Domestic Credit to Private Sector	CR5	Boone Indicator	Lerner Index	Property Rights Index	HHI
***China***										
Mean	0.265	0.049	11.091	0.506	1.107	0.692	-0.020	0.363	27.143	0.178
Standard Deviation	0.244	0.040	1.474	0.398	0.122	0.050	0.008	0.095	4.525	0.025
Minimum	-0.097	0.001	6.402	0.057	0.845	0.578	-0.029	0.206	20.000	0.138
Maximum	3.063	0.179	14.334	1.767	1.276	0.769	-0.004	0.546	30.000	0.218
**India**										
Mean	0.146	0.049	9.228	0.507	0.349	0.433	-0.070	0.221	50.000	0.087
Standard Deviation	0.198	0.049	1.264	0.261	0.102	0.022	0.015	0.056	0.000	0.004
Minimum	-0.373	0.002	5.644	0.230	0.221	0.395	-0.095	0.139	50.000	0.079
Maximum	1.942	0.214	12.175	1.098	0.499	0.470	-0.044	0.314	50.000	0.094
**Indonesia**										
Mean	0.243	0.053	8.119	0.275	0.322	0.611	-0.039	0.147	37.229	0.124
Standard Deviation	1.034	0.048	1.243	0.088	0.137	0.080	0.016	0.057	9.623	0.037
Minimum	-0.907	0.000	2.398	0.140	0.199	0.499	-0.063	0.042	30.000	0.080
Maximum	1.182	0.252	11.229	0.450	0.608	0.766	-0.019	0.235	50.000	0.190
**Japan**										
Mean	-0.008	0.050	11.285	0.747	1.943	0.497	-0.019	0.201	79.412	0.099
Standard Deviation	0.117	0.047	1.131	0.152	0.171	0.072	0.006	0.124	9.389	0.014
Minimum	-0.406	0.002	8.431	0.541	1.748	0.378	-0.024	-0.127	70.000	0.076
Maximum	0.897	0.224	13.240	1.060	2.278	0.588	-0.002	0.426	90.000	0.118
**Korea**										
Mean	0.099	0.055	10.020	0.588	1.105	0.934	-0.093	0.292	79.412	0.150
Standard Deviation	0.643	0.083	1.196	0.260	0.361	0.087	0.070	0.048	9.997	0.055
Minimum	-0.896	0.001	7.789	0.160	0.533	0.738	-0.201	0.176	70.000	0.040
Maximum	1.736	1.131	13.905	0.999	1.596	1.000	-0.020	0.350	90.000	0.200
**Malaysia**										
Mean	0.090	0.182	8.252	1.496	1.231	0.694	-0.035	0.288	57.353	0.152
Standard Deviation	0.253	0.566	1.306	0.416	0.189	0.182	0.010	0.154	9.426	0.046
Minimum	-0.760	0.001	4.890	1.071	0.967	0.398	-0.054	-0.012	50.000	0.055
Maximum	3.745	5.248	11.043	2.622	1.585	0.890	-0.020	0.520	70.000	0.220
**Philippine**										
Mean	0.126	0.053	7.041	0.512	0.356	0.781	-0.228	0.139	46.471	0.148
Standard Deviation	0.283	0.076	1.218	0.162	0.076	0.129	0.167	0.116	17.155	0.036
Minimum	-0.625	0.001	2.833	0.283	0.287	0.571	-0.509	-0.153	30.000	0.080
Maximum	4.176	0.460	10.043	0.846	0.565	0.944	-0.054	0.266	70.000	0.198
**Hong Kong**										
Mean	0.231	0.052	8.645	0.400	0.252	0.498	-0.038	0.171	37.353	0.104
Standard Deviation	0.372	0.067	1.539	0.263	0.133	0.169	0.013	0.074	10.741	0.032
Minimum	-0.575	0.002	4.866	0.016	0.083	0.297	-0.073	0.041	25.000	0.059
Maximum	2.963	0.300	12.185	1.009	0.462	0.792	-0.014	0.276	50.000	0.158
**Singapore**										
Mean	0.058	0.059	7.417	1.666	0.992	0.962	-0.029	0.258	90.000	0.373
Standard Deviation	0.196	0.122	1.749	0.382	0.077	0.041	0.033	0.133	0.000	0.089
Minimum	-0.587	0.000	3.689	1.016	0.860	0.866	-0.127	0.038	90.000	0.269
Maximum	1.639	0.888	11.239	2.428	1.178	1.014	0.000	0.484	90.000	0.500
**Thailand**										
Mean	0.142	0.047	8.398	0.550	1.208	0.661	-0.048	0.086	63.529	0.111
Standard Deviation	0.340	0.049	1.075	0.190	0.215	0.018	0.009	0.223	15.625	0.007
Minimum	-0.653	0.003	5.513	0.238	0.952	0.630	-0.062	-0.455	45.000	0.102
Maximum	4.212	0.279	11.140	0.820	1.657	0.700	-0.035	0.320	90.000	0.128

Note: Table reports country wise averages, standard deviation, and minimum and maximum values on important variables.

Two competition measures (the Lerner Index and Boone Indicator) represent the level of competition in banking markets. However, the ranking of countries with respect to the Lerner Index and Boone Indicator are slightly different. For example, China has the least competitive banking market in terms of the Lerner Index (0.363), but it is the second least competitive in term of the Boone Indicator (-0.020). Similarly, Japan is the least competitive in terms of the Boone Indicator, with an average value of -0.019; however, it is 5^th^ on the level of competition when measured through the Lerner Index.

In terms of industry growth, China is at the top, followed by Indonesia, Hong Kong, India, Thailand and the Philippines, with average values of industrial growth at 0.265, 0.243, 0.231, 0.146, 0.142 and 0.126 respectively. Financial development is estimated using two indices: market capitalization, which measures capital market development, and domestic credit to the private sector, which measures bank development. In terms of bank development, Japan is at the top, followed by Malaysia, Thailand, China and Korea with average values on bank development at 1.943, 1.231, 1.208, 1.107 and 1.1.5 respectively.

Correlations among important variables are reported in Table 3. There are two important considerations with respect to correlations among independent variables. First, that correlations among independent variables are not so high that they create the problem of multicollinearity. Second, that dependent variable has a significant relationship with explanatory variables, especially the variables of interest. Signs and magnitude with respect to the second consideration are not important at this stage because simple correlation may not depict the true relationship without controlling for other relevant explanatory variables. A few high correlations in Table 3 do raise concern about the issue of multicollinearity (i.e. 0.806, 0.652, and 0.632). However, some variables such as CR and HHI are used alternatively in the model and do not appear together. We follow [54] to handle this issue by taking the lag values for highly correlated variables when they are used together. We also apply the estimation technique with and without such variables to observe the differences. However, the results are qualitatively similar. Column 1 of Table 3 shows the correlation between our dependent variable and explanatory ones. Most of these correlations are significant, with expected signs, and represent a rough picture of relationships among variables of interest. Nevertheless, it is too early to draw conclusions on the basis of simple correlations.

**Table 3:** Correlation Matrix.

Names of Variables	(1)	(2)	(3)	(4)	(5)	(6)	(7)	(8)	(9)	(10)	(11)	(12)	(13)	(14)
**(1) Growth in Value Added**	1													
**(2) Industry Share of VA**	.024**	1												
**(3) Financial Dependence**	.038*	.077**	1											
**(4) Market Capitalization/GDP**	-.079**	.164**	.007	1										
**(5) Domestic Credit to Private Sector**	-.104**	.070**	.000	.383**	1									
**(6) CR5**	-.033**	-.074**	.006	.246**	.051**	1								
**(7) Boon Indicator**	.015**	.011**	.005	.161**	.306**	-.263**	1							
**(8) Foreign Bank Share**	.031**	.090**	-.012	.067**	-.118**	.112**	.124**	1						
**(9) Log of Per Capita GDP**	-.122**	.015**	.007	.497**	.652**	.302**	.272**	.040*	1					
**(10) Lerner Index**	.029**	.032**	.005	.235**	.175**	.106**	.150**	.074**	.141**	1				
**(11) Growth in Industry VA**	.049**	.021**	.003	.146**	-.113**	.072**	.069**	.130**	-.138**	.263**	1			
**(12) Property Rights**	-.153**	.044*	.003	.411**	.516**	.394**	-.108**	-.092**	.671**	-.041*	-.157**	1		
**(13) HHI**	-.043**	-.027**	.016	.463**	.113**	.634**	.059**	-.133**	.343**	.246**	.081**	.369**	1	
**(14) GDP Growth**	.046**	.004**	.004	.207**	-.177**	.023	.029	.013	-.208**	.320**	.806**	-.204**	.060**	1

Note: Table reports pairwise correlation among the variables of the study. Indicators “**” and “*” show the statistical significance of correlations at 1% and 5% levels respectively. Description and sources of data are presented in Table 1.

### Results and Discussion

#### Basic Model

In the first step, we explore the role of concentration/competition for industrial growth in general, regardless of specific industry characteristics (i.e. external financial dependence). Tables 4 and 5 report the results of estimation based on Eq 1. The dependent variable in all the specifications is the annual growth of real value added of the manufacturing industries. Two indicators (CR5 and HHI) are used as the measure of bank concentration while the Lerner Index and Boone Indicator measure bank competition. Following [20], we also include a log of per capita GDP, domestic credit to the private sector, market capitalization, and depth of credit information.

**Table 4:** Concentration and Industrial Growth.

Dependent Variable in all specifications is annual growth in real Value Added				
	CR5		HHI	
	(1)	(2)	(3)	(4)
Market Structure (Concentration)	-0.461***	-0.423***	-0.297**	-0.271**
	(0.167)	(0.162)	(0.129)	(0.116)
Domestic Credit to Private Sector	-	0.411***	-	0.394***
	-	(0.132)	-	(0.129)
Market Capitalization to GDP	-	0.215***	-	0.221***
	-	(0.0716)	-	(0.0712)
Accounting Standards	-	0.0083**	-	0.0181**
	-	(0.0037)	-	(0.007)
Log of Per Capita GDP	-	-1.311***	-	-1.781***
	-	(0.485)	-	(0.659)
Industry Share of Value Added	-	-0.259**	-	-0.301**
	-	(0.123)	-	(0.127)
Observations	3,367	3,367	3,367	3,367
R-squared	0.372	0.679	0.397	0.713
Time Dummy	Yes	Yes	Yes	Yes
Industry Dummy	Yes	Yes	Yes	Yes
Country Dummy	Yes	Yes	Yes	Yes

Note: The table displays the impact of market structure (concentration) along with other variables on growth in industry value added. The dependent variable in all the specifications is the annual growth rate of real value added of the manufacturing industries. The main regressors are the 5-bank concentration ratio (CR5) and the Herfindahl-Hirschman Index (HHI), which represent the bank market structure (Bank Concentration). Columns 1 and 3 report the result of estimation when CR5 and HHI respectively are used as the main regressors without including other controls in the model. Columns 3 and 5 show the estimation results when models are run considering other relevant variables. Domestic credit to the private sector as a fraction of GDP proxies for the banking sector development. Market capitalization as a fraction of GDP measures the capital market development. All regressions include country, industry and time dummies in order to tackle unobserved heterogeneity. Robust standard errors are in parentheses, ***, **, and * show the significance at 1%, 5%, and 10% respectively.

**Table 5:** Competition and Industrial Growth.

Dependent Variable in all specifications is annual growth in real Value Added				
	Lerner Index		Boone Indicator	
	(1)	(2)	(3)	(4)
Market Structure (Competition)	-0.319**	-0.285**	-0.293**	-0.279**
	(0.144)	(0.123)	(0.127)	(0.119)
Log of Per Capita GDP	-	-1.564***	-	-1.562***
	-	(0.558)	-	(0.591)
Domestic Credit to Private Sector	-	0.346***	-	0.358***
	-	(0.114)	-	(0.118)
Market Capitalization to GDP	-	0.173***	-	0.151***
	-	(0.0576)	-	(0.0471)
Accounting Standards	-	0.0168**	-	0.0171**
	-	(0.008)	-	(0.007)
Industry Share of Value Added	-	0.312**	-	0.322**
	-	(0.147)	-	(0.147)
Observations	3,367	3,367	3,367	3,367
R-squared	0.377	0.684	0.391	0.692
Time Dummy	Yes	Yes	Yes	Yes
Industry Dummy	Yes	Yes	Yes	Yes
Country Dummy	Yes	Yes	Yes	Yes

Note: The table displays the impact of market structure (competition) along with other variables on industry value added. The dependent variable in all specifications is the annual growth rate of real value added of manufacturing industries. The main regressors are the Lerner Index and Boone Indicator, which represent bank competition. Columns 1 and 3 report the results of estimation when Lerner and Boone are used as the main regressors without including other controls in the model. Columns 3 and 5 show the estimation results when models are run considering other relevant variables. Domestic credit to the private sector as a fraction of GDP proxies for banking sector development. Market capitalization as a fraction of GDP measures the capital market development. All regressions include country, industry and time dummies in order to tackle unobserved heterogeneity. Robust standard errors in parentheses, ***, **, and * show the significance at 1%, 5%, and 10% respectively.

Columns 1 and 3 in Table 4 display the estimation results when CR5 and HHI are used respectively as the main regressors without controlling for other factors. In Columns 2 and 4 we report the estimation results when other factors have also been accounted for. The coefficients on both of the concentration measures are consistently significant, with negative signs. These results imply that a rise in bank concentration in general has detrimental effects on industrial growth. The economic significance of coefficients is also important here. One percentage point increase in the level of concentration may lead to a decrease of around 0.46 percent in industrial growth. These findings concur with those of [20] who also find that bank concentration in general slows down industrial growth. [4] and [48] do not report coefficients on measures of concentration, but only discuss the interaction terms. However, similar results can be inferred from [4]. [48] and [50] provide contradictory findings with respect to the impact of concentration on economic growth. Table 5 reports the estimation results when two competition measures (Lerner Index and Boone Indicator) have been used as the main regressors.

Columns 1 and 3 in Table 5 display the estimation results when competition measures have been used as the main regressors excluding the other factors. Columns 2 and 4 show the estimation results when other factors have also been included in the model. In all the estimations, the coefficients on both Lerner and Boone are significant with negative signs. Interpretation of negative signs for Lerner and Boone is bit tricky. Since the larger values of the Lerner Index indicate more market power and less competition, the negative coefficient for the Lerner Index reinforces our findings in Table 4 that a low level of competition in the banking industry depresses industrial growth. Similarly, the smaller values of the Boone Indicator (higher values with negative signs) indicate a higher level of competition, and therefore the negative coefficient on Boone implies that a low level of competition undermines industrial growth. Our findings for the relationship between competition industrial growth are in agreement with [4] and [50]. However, [48] finds a low level of competition to be favorable for industrial growth.

The behavior of coefficients in relation to other variables is in agreement with the literature: for example, domestic credit to the private sector which proxies for bank development has positive and significant coefficient, indicating that industries with better developed banking systems exhibit higher growth. Per capita GDP, which shows the convergence effect of the economy to its long run equilibrium, has negative and significant coefficient as expected. Market capitalization, which represents development of capital markets, has positive coefficient showing that industries grow more in economies where capital markets are more developed. The coefficient on the accounting standards index is positive, showing that more disclosure enables industries to grow more, as they are able to obtain finances from a variety of investors. These findings conform to earlier studies such as [20], [4] and [48]. These findings suggest that higher bank concentration is likely to slow down the industrial growth in general, whereas the bank competition encourages the growth of manufacturing industries.

#### Extended Model

Table 6 reports the results of an estimation based on Eq 2, where we include two interaction terms (the interaction between external dependence and concentration/competition, and the interaction between external dependence and financial development). Both financial dependence and financial development have significant and positive coefficients, suggesting that industries which are dependent on external finance and those operating in well-developed financial systems grow more. The coefficients on all market structure measures maintain their significance with expected signs. The interaction terms between concentration (both CR5 and HHI) and financial dependence is negative and significant, implying that bank concentration shrinks the growth of externally financially dependent industries. These finding are in contrast to earlier evidence provided by [20] and [48], who show a positive relationship between bank concentration and the growth of externally financially dependent industries. On the other hand, [4] provide weak evidence of a negative impact of bank concentration on the growth of externally financially dependent industries.

**Table 6:** Concentration, Competition, Financial Development, Financial Dependence and Industrial Growth.

Dependent Variable in all specifications is annual growth in real Value Added				
	Concentration		Competition	
	(1)	(2)	(3)	(4)
Market Structure	-0.396***	-0.262**	-0.251**	-0.233**
	(0.142)	(0.118)	(0.109)	(0.104)
Market Structure*Financial Dependence	-0.0615***	-0.0551**	-0.0525**	-0.0547**
	(0.022)	(0.023)	(0.021)	(0.024)
Financial Dependence	0.071**	0.093**	0.0821**	0.0802**
	(0.031)	(0.038)	(0.035)	(0.033)
Financial Development	0.971***	0.946***	0.983***	0.974***
	(0.322)	(0.293)	(0.313)	(0.318)
Financial Dependence*Financial Development	0.0811**	0.0871**	0.0682**	0.0726**
	(0.033)	(0.036)	(0.029)	(0.031)
Log of Per Capita GDP	-1.347***	-1.358***	-1.361***	-1.359***
	(0.471)	(0.441)	(0.393)	(0.419)
Industry Share of Value Added	-0.038**	-0.065**	-0.046**	-0.039**
	(0.017)	(0.029)	(0.021)	(0.018)
Observations	3,168	3,168	3,168	3,168
R-squared	0.697	0.726	0.715	0.711
Time Dummy	Yes	Yes	Yes	Yes
Country Dummy	Yes	Yes	Yes	Yes
Industry Dummy	Yes	Yes	Yes	Yes

Note: The table reports the results of estimation when interaction terms between sectoral financial dependence and market structure (concentration and competition) and country level financial development are included in the model. The dependent variable in all specifications is the annual growth rate of real value added of manufacturing industries. Columns 1 and 2 report the results of estimation from the 5-bank concentration ratio (CR5) and the Herfindahl-Hirschman Index (HHI) respectively. CR5 and HHI both represent the bank concentration. Columns 3 and 4 show the estimation results from the Lerner Index and Boone Indicator respectively, both of which measure the banking competition. Domestic credit to the private sector as a fraction of GDP proxies for banking sector development. Market capitalization as fraction of GDP measures the capital market development. All regressions include country, industry and time dummies in order to tackle unobserved heterogeneity. Robust standard errors in parentheses, ***, **, and * show the significance at 1%, 5%, and 10% respectively.

The coefficient on the interaction term between competition measures (Lerner Index and Boone Indicator) and financial dependence is significant with negative sign. These results suggest that bank competition encourages the growth of externally financial dependent industries. Our results for competition are in agreement with the findings of [4], who find that bank competition has a positive impact on the growth of externally financially dependent industries. [48], however, shows that bank competition is negatively related to the growth of industries that depend on external finance; this is in contrast to both [4] findings and our own. The interaction term between external dependence and financial development enters the model with positive and significant coefficient, indicating that externally financially dependent industries grow more in financially developed economies. Our findings for financial development and financial dependence are in agreement with earlier literature [4, 20, 40–42, 48, 55]. Other variables such as per capita GDP have expected and consistent signs in all specifications.

### Robustness Check

In this section we conduct a number of robustness checks to ensure that the results reported in Table 6 reflect the true relationship between market structure and the growth of externally financially dependent industries. We specifically account for growth opportunities, property rights, accounting standards, foreign bank ownership and endogeneity.

#### Growth opportunities

It is quite possible that the relationship between industrial growth and financial development is driven by other elements that are also responsible for industrial growth across the economies. For instance, [53] maintain that growth rate differentials across the economies are better explained by the presence of growth opportunities rather than financial development. A similar argument may also apply to competition/concentration. In other words, it is not the availability/unavailability of finance to externally dependent industries in a competitive/ concentrated banking system that leads to sectoral growth, but rather the existence/non-existence of growth prospects which somehow are related to concentration/competition. Following [4] and [53], we use industry sales (Data on industry sales come from UNIDO) as a proxy for growth opportunities. We include interaction terms of growth opportunities with concentration, competition, financial dependence and financial development. The estimation results for growth opportunities are reported in Table 7. Alternatively, we also use price-earnings ratio (a market based measure) to capture the effect of growth opportunities. Result for price-earnings ratio are reported in Table 8. The coefficients on the interaction terms of financial development and growth opportunities are positive and significant; however, the interaction term of financial development and financial dependence in not significant in some cases. These findings are similar to those of [4] and [53]. What is important for our study is the finding that the interaction terms of financial dependence and market structure measures (concentration and competition) have expected and significant coefficients, implying that even after accounting for growth opportunities, bank concentration seems to suppress the growth of externally financially dependent industries while more competitive banking systems are likely allow these industries to grow more.

**Table 7:** Accounting for Growth Opportunities (by Industrial Sales).

Dependent Variable in all specifications is annual growth in real Value Added				
	Concentration		Competition	
	(1)	(2)	(3)	(4)
Market Structure*Financial Dependence	-0.092**	-0.095***	-0.119**	-0.128***
	(0.037)	(0.027)	(0.049)	(0.041)
Market Structure*Growth Opportunities	-0.982	-0.971	-0.917	-0.751
	(0.545)	(0.693)	(0.611)	(0.536)
Financial Development*Growth Opportunities	0.641**	0.593**	0.476**	0.528**
	(0.268)	(0.257)	(0.193)	(0.219)
Financial Dependence*Financial Development	0.552	0.539	0.573	0.541
	(0.324)	(0.362)	(0.411)	(0.337)
Industry Share of Value Added	-0.107**	-0.109**	-0.142**	-0.143**
	(0.005)	(0.005)	(0.006)	(0.006)
Observations	3,367	3,367	3,367	3,367
R-squared	0.197	0.199	0.218	0.221
Time Dummy	Yes	Yes	Yes	Yes
Country Dummy	Yes	Yes	Yes	Yes
Industry Dummy	Yes	Yes	Yes	Yes

Note: The table shows the robustness of results by incorporating the growth opportunities into estimation results reported in Table 6. The dependent variable in all specifications is the annual growth rate of real value added of the manufacturing industries. Columns 1 and 2 report the result of estimation from the 5-bank concentration ratio (CR5) and the Herfindahl-Hirschman Index (HHI) respectively, both of which represent the bank concentration. Columns 3 and 4 show the estimation results from the Lerner Index and Boone Indicator respectively, both of which measure banking competition. Domestic credit to the private sector as a fraction of GDP proxies for banking sector development. Market capitalization as a fraction of GDP measures capital market development. All regressions include country, industry and time dummies in order to tackle unobserved heterogeneity. Robust standard errors in parentheses, ***, **, and * show the significance at 1%, 5%, and 10% respectively.

**Table 8:** Accounting for Growth Opportunities (by Price-Earnings Ratio).

Dependent Variable in all specifications is annual growth in real Value Added				
	Concentration		Competition	
	(1)	(2)	(3)	(4)
Market Structure*Financial Dependence	-0.087**	-0.113***	-0.093**	-0.078***
	(0.033)	(0.032)	(0.041)	(0.022)
Market Structure*Growth Opportunities (P/E)	-0.042*	-0.073**	-0.117	-0.057
	(0.015)	(0.35)	(0.216)	(0.536)
Financial Development*Growth Opportunities (P/E)	0.473**	0.439**	0.361**	0.454**
	(0.217)	(0.173)	(0.178)	(0.223)
Financial Dependence*Financial Development	0.452	0.437*	0.497*	0.443
	(0.324)	(0.224)	(0.251)	(0.337)
Industry Share of Value Added	-0.172**	-0.193**	-0.127**	-0.138**
	(0.081)	(0.096)	(0.061)	(0.067)
Observations	3,367	3,367	3,367	3,367
R-squared	0.276	0.298	0.322	0.329
Time Dummy	Yes	Yes	Yes	Yes
Country Dummy	Yes	Yes	Yes	Yes
Industry Dummy	Yes	Yes	Yes	Yes

Note: The table shows the robustness of results by incorporating the growth opportunities (price earnings ratio) into estimation results reported in Table 6. The dependent variable in all specifications is the annual growth rate of real value added of the manufacturing industries. Columns 1 and 2 report the result of estimation from the 5-bank concentration ratio (CR5) and the Herfindahl-Hirschman Index (HHI) respectively, both of which represent the bank concentration. Columns 3 and 4 show the estimation results from the Lerner Index and Boone Indicator respectively, both of which measure banking competition. Domestic credit to the private sector as a fraction of GDP proxies for banking sector development. Market capitalization as a fraction of GDP measures capital market development. All regressions include country, industry and time dummies in order to tackle unobserved heterogeneity. Robust standard errors in parentheses, ***, **, and * show the significance at 1%, 5%, and 10% respectively.

#### Property Rights

The economic literature suggests that institutional factors such as enforcement of property rights play an important role in industrial growth and financial development (see for example, [56]; and [57]. In order to ensure that our findings are not influenced by such institutional characteristics, we follow [4] and include the interaction between property rights and financial dependence. The results from this analysis are shown in Table 9. The interaction term of property rights and financial dependence enters with positive and significant coefficient, indicating that financially dependent industries grow more in economies with a greater enforcement of property rights. Importantly, the coefficients on the interaction term between market structure (competition and concentration) and financial dependence are still significant and with consistent signs. Thus even after controlling for property rights, our main findings are unchanged: in other words, a higher level of competition and a lower level of concentration in banking industry foster the growth of financially dependent industries.

**Table 9:** Accounting for Property Rights.

Dependent Variable in all specifications is annual growth in real Value Added				
	Concentration		Competition	
	(1)	(2)	(3)	(4)
Market Structure*Financial Dependence	-0.127**	-0.213***	-0.257***	-0.296**
	(0.051)	(0.073)	(0.079)	(0.123)
Financial Dependence*Financial Development	0.617***	0.609***	0.625***	0.613***
	(0.114)	(0.191)	(0.211)	(0.181)
Property Rights*Financial Dependence	0.0317***	0.0336***	0.0352***	0.0358***
	(0.012)	(0.009)	(0.008)	(0.011)
Industry Share of Value Added	-0.381**	-0.448*	-0.448*	-0.447*
	(0.181)	(0.266)	(0.264)	(0.264)
Observations	3,367	3,367	3,367	3,367
R-squared	0.297	0.314	0.322	0.331
Time Dummy	Yes	Yes	Yes	Yes
Country Dummy	Yes	Yes	Yes	Yes
Industry Dummy	Yes	Yes	Yes	Yes

Note: The table displays the robustness of results by incorporating the property rights into the estimation results reported in Table 6. The dependent variable in all specifications is the annual growth rate of real value added of the manufacturing industries. Columns 1 and 2 report the result of the estimation from the 5-bank concentration ratio (CR5) and the Herfindahl-Hirschman Index (HHI) respectively, both of which represent the bank concentration. Columns 3 and 4 show the estimation results from the Lerner Index and Boone Indicator respectively, both of which measure banking competition. Domestic credit to the private sector as a fraction of GDP proxies for banking sector development. Market capitalization as a fraction of GDP measures capital market development. All regressions include country, industry and time dummies in order to tackle unobserved heterogeneity. Robust standard errors in parentheses, ***, **, and * show the significance at 1%, 5%, and 10% respectively.

#### Accounting Standards

Economic growth across countries may vary because industries have varying access to external finance, depending upon the quality of disclosure. According to [4], “quality of accounting standards” refers to all forms of external financing and not just to banking and stock markets. Moreover, quality of accounting standards reflects the potential to obtain finance. [40] claim that higher standards of financial disclosure enable firms to raise finance from a vast group of investors. We include in our estimation the interaction term of accounting standards with financial dependence, and these results are reported in Table 10. The coefficient on the interaction term between accounting standards and financial dependence is significant and positive. Our findings for accounting standards are in agreement with earlier studies (for example,[4, 20, 40]. What is important for our analysis is that the coefficient on the interaction of financial dependence and financial development is significant. The main findings of our study are reinforced: namely, the interaction term of financial dependence and concentration/competition remained unchanged. Thus our results are not driven by availability of better quality information.

**Table 10:** Controlling for Accounting Standards.

Dependent Variable in all specifications is annual growth in real Value Added				
	Concentration		Competition	
	(1)	(2)	(3)	(4)
Market Structure*Financial Dependence	-0.142**	-0.243***	-0.236***	-0.258***
	(0.061)	(0.072)	(0.069)	(0.077)
Financial Dependence*Financial Development	0.353***	0.396***	0.417***	0.315***
	(0.103)	(0.125)	(0.121)	(0.112)
Accounting Standards*Financial Dependence	0.219***	0.208***	0.203***	0.197***
	(0.063)	(0.067)	(0.061)	(0.059)
Industry Share of Value Added	-0.134**	-0.161**	-0.175**	-0.144**
	(0.067)	(0.079)	(0.083)	(0.065)
Observations	3,367	3,367	3,367	3,367
R-squared	0.261	0.265	0.258	0.272
Time Dummy	Yes	Yes	Yes	Yes
Country Dummy	Yes	Yes	Yes	Yes
Industry Dummy	Yes	Yes	Yes	Yes

Note: The table displays the robustness of results by incorporating quality of accounting standards into the estimation results reported in Table 6. The dependent variable in all specifications is the annual growth rate of real value added of manufacturing industries. Columns 1 and 2 report the result of estimation from the 5-bank concentration ratio (CR5) and Herfindahl-Hirschman Index (HHI) respectively, both of which represent bank concentration. Columns 3 and 4 show the estimation results from the Lerner Index and Boone Indicator respectively, both of which measure banking competition. Domestic credit to the private sector as a fraction of GDP proxies for banking sector development. Market capitalization as a fraction of GDP measures the capital market development. All regressions include country, industry and time dummies in order to tackle unobserved heterogeneity. Robust standard errors in parentheses, ***, **, and * show the significance at 1%, 5%, and 10% respectively.

#### Bank Ownership

Industrial growth may be different across countries not only because of banking concentration/competition but also because of the ownership structure of the banking sectors. For example, firms’ access to credit in economies dominated by state-owned banks can be lower because it is possible that such banks do not have enough incentives to build strong lending relationships with successful ventures [20]. In contrast, the existence of foreign banks can increase competition and improve efficiency, which ultimately increases the credit supply [58–61]. Similarly, a reduced cost of borrowing as a result of foreign acquisitions can lead to firms having greater access to credit [62]. Nevertheless, the pressure from foreign banks can cause domestic banks to reduce the credit supply, thus firms’ access to credit decreases [63–65].

We use data on the share of foreign and state owned banks to examine their role in the relationship between concentration/competition and industrial growth. Table 11 shows the results of estimation where the interaction terms of foreign owned banks and state owned banks with financial dependence have been used (Data on foreign and state owned banks’ share comes from different sources, such as the central banks of sample countries, Helgi Library and the World Bank). The coefficient on the interaction term between financial dependence and state owned banks is negative and significant, thus supporting the argument that a higher share of state owned banks depresses industrial growth. The coefficient ton the interaction term between foreign banks and financial dependence is positive and significant, implying that a higher share foreign banks increases the access to credit and therefore fosters the growth of financially dependent industries. Our findings for state owned banks are in agreement with [48]; however, for foreign banks our results contrast with his findings.

**Table 11:** Accounting for Foreign Ownership.

Dependent Variable in all specifications is annual growth in real Value Added				
	Concentration		Competition	
	(1)	(2)	(3)	(4)
Market Structure*Financial Dependence	-0.157**	-0.192**	-0.186**	-0.292***
	(0.071)	(0.087)	(0.081)	(0.126)
Financial Dependence*Financial Development	0.488***	0.516***	0.525***	0.483***
	(0.143)	(0.151)	(0.162)	(0.139)
State Owned Banks’ Share*Financial Development	-0.0474**	-0.0751**	-0.0679**	-0.0691**
	(0.0134)	(0.0221)	(0.0203)	(0.0213)
Foreign Banks’ Share*Financial Dependence	0.316***	0.429***	0.383***	0.379***
	(0.115)	(0.126)	(0.112)	(0.105)
Industry Share of Value Added	0.176**	0.111**	0.157**	0.162**
	(0.081)	(0.051)	(0.071)	(0.076)
Observations	3,367	3,367	3,367	3,367
R-squared	0.316	0.329	0.337	0.318
Time Dummy	Yes	Yes	Yes	Yes
Country Dummy	Yes	Yes	Yes	Yes
Industry Dummy	Yes	Yes	Yes	Yes

Note: The table displays the robustness of results by incorporating the government and foreign banks’ share into the estimation results reported in Table 6. The dependent variable in all specifications is the annual growth rate of real value added of manufacturing industries. Columns 1 and 2 report the result of estimation from the 5-bank concentration ratio (CR5) and Herfindahl-Hirschman Index (HHI) respectively, both of which represent bank concentration. Columns 3 and 4 show the estimation results from the Lerner Index and Boone Indicator respectively, both of which measure banking competition. Domestic credit to the private sector as a fraction of GDP proxies for banking sector development. Market capitalization as a fraction of GDP measures capital market development. All regressions include country, industry and time dummies in order to tackle unobserved heterogeneity. Robust standard errors in parentheses, ***, **, and * show the significance at 1%, 5%, and 10% respectively

Importantly, our main findings (the concentration/competition and growth of financially dependent industries), remain the same, as the coefficients on interaction between the measures of market structure (concentration/competition) and financial dependence remain significant with consistent signs even after controlling for banks’ ownership.

#### Dealing with Endogeneity

According to [20], it is possible that the relationship between market structure and industrial growth suffers from an endogeneity bias because the bank market structure may adjust to the industrial structure. [4] also discuss the possibility of an endogeneity problem in their study. Following [54] and [4], we deal with endogeneity problem by estimating the results with instrumental variable (IV) approach and the Two-step System GMM dynamic panel model with [66] corrected standard errors and small sample adjustments. The results from GMM and the IV approach are reported in Tables 12 and 13 respectively. The coefficients on interaction terms between the measure of market structure (concentration/competition) and financial dependence remain unchanged. Also the coefficients on all the other variables are significant and with expected signs. These results reaffirm our findings that externally financially dependent firms grow more/less in competitive/concentrated banking systems.

**Table 12:** Generalized Method of Moments (GMM) Approach.

Dependent Variable in all specifications is annual growth of industry real Value Added				
	Concentration		Competition	
	(1)	(2)	(3)	(4)
Market Structure	-0.367***	-0.273**	-0.298**	-0.259**
	(0.118)	(0.114)	(0.123)	(0.117)
Market Structure*Financial Dependence	-0.097***	-0.131***	-0.084***	-0.091**
	(0.027)	(0.042)	(0.023)	(0.039)
Financial Dependence	0.087**	0.079**	0.091**	0.089**
	(0.037)	(0.032)	(0.041)	(0.039)
Financial Development	0.773***	0.749***	0.781***	0.774***
	(0.349)	(0.219)	(0.223)	(0.242)
Financial Dependence*Financial Development	0.179**	0.183**	0.187**	0.181***
	(0.074)	(0.079)	(0.084)	(0.058)
Log of Per Capita GDP	-0.0127*	-0.0143**	-0.0129*	-0.0131**
	(0.0067)	(0.0061)	(0.0053)	(0.0056)
Industry Share of Value Added	-0.169**	-0.163**	-0.211**	-0.213**
	(0.079)	(0.077)	(0.095)	(0.096)
AR(1) P value	0.023	0.019	0.027	0.012
AR(2) P value	0.267	0.242	0.341	0.283
Sargan/Hensen P value	0.212	0.181	0.175	0.228
Observations	3,168	3,168	3,168	3,168
Number of Instruments	142	134	158	162
Number of ID	199	199	199	199

Note: The table shows the results from the Two step system GMM when applied to the model in Equation 3.2. The dependent variable in all specifications is the annual growth rate of real value added of manufacturing industries. Columns 1 and 2 report the result of estimation from the 5-bank concentration ratio (CR5) and the Herfindahl-Hirschman Index (HHI) respectively, both of which represent bank concentration. Columns 3 and 4 show the estimation results from the Lerner Index and Boone Indicator respectively, both of which measure banking competition. Domestic credit to the private sector as a fraction of GDP proxies for banking sector development. Market capitalization as a fraction of GDP capital market development. All regressions include country, industry and time dummies in order to tackle unobserved heterogeneity. Robust standard errors in parentheses, ***, **, and * show the significance at 1%, 5%, and 10% respectively.

**Table 13:** The Instrumental Variable (IV) Approach.

Dependent Variable in all specifications is annual growth in real Value Added				
	Concentration		Competition	
	(1)	(2)	(3)	(4)
Market Structure	-0.285**	-0.213**	-0.239**	-0.199**
	(0.138)	(0.108)	(0.118)	(0.098)
Market Structure*Financial Dependence	-0.081***	-0.063**	-0.071**	-0.077**
	(0.023)	(0.029)	(0.033)	(0.036)
Financial Dependence	0.109**	0.132**	0.121**	0.102**
	(0.052)	(0.058)	(0.055)	(0.047)
Financial Development	0.673***	0.648***	0.685***	0.676***
	(0.213)	(0.201)	(0.221)	(0.224)
Financial Dependence*Financial Development	0.182**	0.179**	0.168**	0.171**
	(0.083)	(0.077)	(0.071)	(0.081)
Log of Per Capita GDP	-0.437**	-0.538**	-0.631**	-0.593**
	(0.211)	(0.259)	(0.309)	(0.281)
Industry Share of Value Added	-0.081**	-0.098**	-0.079**	-0.095**
	(0.037)	(0.043)	(0.035)	(0.046)
Observations	3,168	3,168	3,168	3,168
R-squared	0.671	0.712	0.693	0.639
Time Dummy	Yes	Yes	Yes	Yes
Country Dummy	Yes	Yes	Yes	Yes
Industry Dummy	Yes	Yes	Yes	Yes

Note: The table reports the results from the instrumental variable (IV) approach. The dependent variable in all specifications is the annual growth rate of real value added of manufacturing industries. Columns 1 and 2 report the results of estimation from the 5-bank concentration ratio (CR5) and the Herfindahl-Hirschman Index (HHI) respectively. CR5 and HHI both represent the bank concentration. Columns 3 and 4 show the estimation results from the Lerner Index and Boone Indicator respectively, both of which measure the banking competition. Domestic credit to the private sector as a fraction of GDP proxies for banking sector development. Market capitalization as fraction of GDP measures the capital market development. All regressions include country, industry and time dummies in order to tackle unobserved heterogeneity. Robust standard errors in parentheses, ***, **, and * show the significance at 1%, 5%, and 10% respectively.

#### Market Structure-Industrial Growth Relationship and the Financial Crisis

Although, the fluctuations in industrial growth in pre and post financial crisis periods have been accounted for by introducing country, industry and time fixed effects, as an additional robustness check, the analysis has also been performed on different sample periods (we are thankful to anonymous referee for this valuable insight) i.e. subsample 1996–1999 (Asian financial crises period), subsample 2000–2006 (post financial crisis period) and subsample 2007–2010 (Global financial crisis period). Sample has been divided in three groups on the basis of (i) insights from earlier studies [i.e. [67–69]] and (ii) coefficients on time dummies for year 1996, 1997, 1998, 1999, 2007, 2008, 2009 and 2010. Although, not reported in table for brevity, the coefficients on these years are significantly negative indicating that industrial growth has been lower during the financial crisis. Table 14 shows the estimation results for subsamples 1996–1999, 2000–2006, and 2007–2010. The analysis has been performed using all measures of market structure, however, we only report results from CR5 and Lerner Index to conserve the space (results from other measures are qualitatively similar to the overall results).

**Table 14:** Market Structure-Industrial Growth Relationship and the Financial Crisis.

Dependent variable: Growth in Industry Value Added						
	Subsample 1996–1999		Subsample 2000–2007		Subsample 2008–2010	
	(1)	(2)	(3)	(4)	(5)	(6)
Market Structure	-0.287**	-0.238**	-0.215***	-0.314***	-0.247*	-0.293**
	(0.141)	(0.117)	(0.071)	(0.082)	(0.131)	(0.137)
Market Structure*Financial Dependence	-0.040**	-0.050**	-0.082**	-0.119***	-0.073**	-0.047*
	(0.019)	(0.021)	(0.039)	(0.037)	(0.032)	(0.026)
Financial Dependence	0.043**	0.039**	0.052***	0.086***	0.049*	0.063**
	(0.021)	(0.019)	(0.011)	(0.027)	(0.026)	(0.031)
Financial Development	0.303***	0.349***	0.314**	0.372**	0.329*	0.355**
	(0.095)	(0.111)	(0.153)	(0.183)	(0.169)	(0.173)
Financial Dependence*Financial Development	0.069**	0.066**	0.063***	0.083**	0.102*	0.098*
	(0.027)	(0.029)	(0.016)	(0.041)	(0.058)	(0.053)
Log of Per Capita GDP	-0.518**	-0.615**	-0.539**	-0.553**	-0.572**	-0.534**
	(0.252)	(0.297)	(0.266)	(0.247)	(0.274)	(0.258)
Industry Share of Value Added	-0.088***	-0.075***	-0.069***	-0.061***	-0.058**	-0.082**
	(0.018)	(0.021)	(0.019)	(0.013)	(0.015)	(0.023)
Observations	920	920	1840	1840	1840	1840
R-squared	(0.498)	(0.532)	(0.571)	(0.551)	(0.517)	(0.546)
Time Dummy	Yes	Yes	Yes	Yes	Yes	Yes
Country Dummy	Yes	Yes	Yes	Yes	Yes	Yes
Industry Dummy	Yes	Yes	Yes	Yes	Yes	Yes

Note: Table reports estimation results of the extended model for different sample periods. Panel A, B and C report the results for Subsample 1996–1999, Subsample 2000–2007 and Subsample 2008–2010 respectively. The dependent variable in all specifications is the annual growth of real value added of manufacturing industries. Column 1, 3 and 5 show the estimation results when CR5 is used as measure of market structure. While, the columns 2, 4 and 6 report the estimation results when Lerner Index is used as measure of market structure. Domestic credit to the private sector as a fraction of GDP proxies for banking sector development. Market capitalization as fraction of GDP measures the capital market development. All regressions include country, industry and time dummies in order to tackle unobserved heterogeneity. Robust standard errors in parentheses, ***, **, and * show the significance at 1%, 5%, and 10% respectively.

Important for this analysis are the coefficients on market structure variables, the interaction between market structure and financial dependence and the interaction between financial development and financial dependence. Coefficients on both CR5 and Lerner Index across all the sample periods are significant with negative sign. Similarly, the coefficients on the interaction terms between market structure and financial dependence, and the financial dependence and financial development are significant with the expected sign. Moreover, the behavior of other variables across all the sample periods is also similar to results from the main estimation. Over all, our findings (externally financially dependent firms grow more/less in competitive/concentrated banking systems) are robust across different sample periods.

## Conclusion

We have studied the role of bank concentration/competition for growth of manufacturing industries that are dependent on external financing. We have taken into account two structural and two non-structural measures of market structure and applied them to industry level data on the manufacturing industries from 10 emerging Asian economies, including China, over the period of 1995–2011.

The results show that bank concentration (as measured by CR5 and HHI) may slow down the growth of externally financially dependent industries. This finding lends support to the idea that firms in a concentrated banking industry have low access to credit, which leads to less economic growth [18, 19], and that higher concentration in banking sectors leads to less new firm creation and less economic growth [45, 46]. On the other hand, bank competition (as measured by the Lerner Index and Boone Indicator) may allow financially dependent industries to grow faster. This finding supports the notion that a higher degree of competition in the banking industry reduces the holdup problems, reduces the cost of intermediation and encourages financially dependent firms to access bank credit [4].

These findings are robust when subjected to a number of sensitivity checks including alternative measures of financial dependence, and other channels that might explain the relationship between market structure (concentration and competition) and the growth of financially dependent industries. Other factors explaining this relationship include institutional ones, such property rights, quality of accounting standards and ownership of banks. In conclusion, our study suggests that industries in need of external finance grow more in a less concentrated and more in competitive banking environment.

### Policy Implications

The study provides important implications for anti-trust policies. For example, in the aftermath of the Asian financial crisis 1997–98 and the global financial crisis 2008–09, there has been an unprecedented increase in bank consolidations. Based on concentration-stability hypothesis, the idea was to strengthen the financial institutions in the events of financial downturns. The concentration-stability hypothesis suggests that larger banks in concentrated banking industries can reduce the financial system risk. However, the literature reveals that concentrated banking industries may prove to be counterproductive for financial stability, bank efficiency, monetary policy transmission and the economic growth. This study provides the evidence of such counterproductive effects of bank concentration on the economic growth of financially dependent industries. Therefore, consolidation policies must be pursued carefully.
