# Correction: Market Structure, Financial Dependence and Industrial Growth: Evidence from the Banking Industry in Emerging Asian Economies

**DOI:** 10.1371/journal.pone.0162999

**Published:** 2016-09-09

**Authors:** Habib Hussain Khan, Rubi Binti Ahmad, Chan Sok Gee

The second author’s name is spelled incorrectly in the published article. The correct name is: Rubi Binti Ahmad. The correct citation is: Khan HH, Ahmad RB, Gee CS (2016) Market Structure, Financial Dependence and Industrial Growth: Evidence from the Banking Industry in Emerging Asian Economies. PLoS ONE 11(8): e0160452. doi:10.1371/journal.pone.0160452.
